# Novel Index Combining Pan-Immune-Inflammatory Index and Hemoglobin Levels (PIV/Hb) Predicts Trismus Rates Efficiently after Chemoradiotherapy in Locally Advanced Nasopharyngeal Cancer

**DOI:** 10.1155/2024/2124006

**Published:** 2024-09-30

**Authors:** Efsun Somay, Busra Yilmaz, Erkan Topkan, Beyza Sirin Ozdemir, Duriye Ozturk, Ali Ayberk Besen, Huseyin Mertsoylu, Ugur Selek

**Affiliations:** ^1^ Department of Oral and Maxillofacial Surgery Faculty of Dentistry Baskent University, Ankara, Türkiye; ^2^ Department of Oral and Maxillofacial Radiology School of Dental Medicine Bahcesehir University, Istanbul, Türkiye; ^3^ Department of Radiation Oncology Faculty of Medicine Baskent University, Adana, Türkiye; ^4^ Clinics of Radiation Oncology Medical Park Hospital, Antalya, Türkiye; ^5^ Department of Radiation Oncology Faculty of Medicine Afyonkarahisar Health Sciences University, Afyonkarahisar, Türkiye; ^6^ Clinics of Medical Oncology Adana Medical Park Hospital, Adana, Türkiye; ^7^ Clinics of Medical Oncology Istinye University Adana Medical Park Hospital, Istanbul, Türkiye; ^8^ Department of Radiation Oncology School of Medicine Koc University, Istanbul, Türkiye

## Abstract

**Purpose:**

To evaluate the predictive potency of a novel index combining the pan-immune-inflammatory index and hemoglobin levels (PIV/Hb) for the prevalence of radiation-induced trismus (RIT) in patients with locally advanced nasopharyngeal cancer (LA-NPC) receiving concurrent chemoradiotherapy (CCRT).

**Methods:**

Data from 228 LA-NPC patients were retrospectively examined. Maximum mouth openings (MMO) were measured to confirm the presence of RIT, defined as MMOs ≤35 mm. Complete blood test results from the first day of CCRT were used to calculate PIV/Hb levels. A potential relationship between pretreatment PIV/Hb and the RIT status was evaluated using receiver operating characteristic (ROC) curve analysis.

**Results:**

Post-CCRT RIT was diagnosed in 20.2% of the patients. The ROC curve analysis determined 68.4 g/dL as the ideal PIV/Hb cutoff that effectively divided patients into two distinct groups (area under the curve: 94.7%; specificity: 86.4%; sensitivity: 87.4%). RIT was significantly more prevalent in the PIV/Hb > 68 group than in the PIV/Hb < 68 group (58.8% vs. 3.8%; *P* < 0.001). Multivariate logistic regression analysis showed that a pre-CCRT PIV > 68 was independently associated with significantly higher rates of RIT.

**Conclusion:**

Higher pretreatment levels of the novel PIV/Hb index predict increased RIT rates following definitive CCRT for LA-NPCs.

## 1. Introduction

Radiotherapy (RT) is essential in treating nasopharyngeal malignancies (NPC), either alone or in conjunction with chemotherapy, depending on the illness's stage, where the surgery's utility is minimal [1]. Despite substantial advancements in diagnosis, treatment, and supportive care, nearly half of all patients are still experiencing different acute and/or chronic side effects [2]. Common and tolerable side effects include xerostomia, dysphagia, weight loss, dental caries, periodontal disease, mucositis, skin changes, and fibrosis. In contrast, incapacitating side effects include fistula formation, myelitis, hearing loss, mucosal fibrosis, periodontitis, tooth loss, osteoradionecrosis, and radiation-induced trismus (RIT) [3].

Radiation-induced fibrosis of the masticatory muscles, temporomandibular joint (TMJ)-related raphes, and synovial fluid may lead to RIT, which affects up to 42% of HNC patients [4]. While the exact mechanism behind RIT is still not fully understood, it is widely recognized that radiation-induced inflammation, endothelial damage, hypoxia, and fibrosis are the main contributors to its development [5]. Initial steps in the cascade of tissue damage include reactive oxygen species generation and double-stranded deoxyribonucleic acid (DNA) damage in irradiated cells [5]. Subsequently, enzymes associated with tissue damage increase oxidative stress in radiation-damaged tissues, leading to tissue ischemia. These events perpetuate local tissue injury and stimulate the production of proinflammatory cytokines and chemokines [6]. Lending credence to these basic mechanisms, recent studies have indicated that biomarkers such as hemoglobin-to-platelet ratio (HPR) and neutrophil-to-platelet ratio (NLR) may play a role in stimulating the production of inflammatory cytokines and chemokines associated with fibrosis in locally advanced nasopharyngeal cancer (LA-NPC) and parotid cancer patients [7, 8].

The pan-immune-inflammation value (PIV) is an innovative and exclusive biomarker that could suggest fibrosis due to its cellular components and possible cellular byproducts [9]. PIV is a comprehensive blood-borne immune and inflammatory biomarker that may indicate systemic inflammation and immunological activity as it incorporates monocytes, platelets, neutrophils, and lymphocytes [9]. Hypoxia is another factor that can lead to inflammation and fibrosis in tissues, such as the components of masticatory apparatus, including the temporomandibular joints (TMJ) [10]. In this regard, low hemoglobin (Hb) levels serve as an indirect local and direct systemic marker for hypoxia, inflammation, and tissue fibrosis, all cumulatively prompting RIT development [11]. Recent clinical studies provided clinical credence to the potential value of pretreatment Hb levels in predicting the RIT rates by showing that patients presenting with Hb levels below 12.0 g/dL have a higher risk of developing RIT (41.9% vs. 7.3%; *P*  <  0.001) after concurrent chemoradiotherapy (CCRT) for locally advanced nasopharyngeal carcinoma (LA-NPC) when compared to their counterparts with Hb levels of Hb ≥ 12.0 g/dL [12].

Despite the presence of evident potential, to the best of our knowledge, no study examined the combined effect of PIV and Hb in predicting the prevalence of RIT in LA-NPC patients treated with radical CCRT. Hence, the present investigation aimed to investigate whether the pretreatment PIV/Hb ratio, a new composite biomarker of immunity, inflammation, and hypoxia, can predict the prevalence of RIT in this patient population.

## 2. Patients and Methods

### 2.1. Ethics, Consent, and Permissions

The Institutional Review Board (project no. DKA 19/39) of the XX Medical Faculty approved a retrospective investigation following the principles of the Helsinki Declaration and its subsequent amendments. Before the commencement of the CCRT, all participants provided written informed consent for data collection and publication of associated outcomes, as required by our institution's policies and procedures.

### 2.2. Study Population

The present investigation was conducted with the collaboration of the Department of Radiation Oncology and Dentistry Clinics of the XXXX Research and Treatment Center, and it was presented in adherence to the STROBE guidelines. The study comprised patients who underwent comprehensive oral and dental examinations before and after CCRT between 2012 and 2022. Patients diagnosed with LA-NPC who did not exhibit any symptoms of TMD before CCRT, as per the current diagnostic criteria (DC) for TMD (DC/TMD), were included in this study. [13] The eligibility criteria for the study required patients to satisfy certain conditions. These conditions included being between the ages of 18 and 80, having a performance status of 0-1 as per the Eastern Cooperative Oncology Group (ECOG), presenting with histopathologic evidence of squamous cell NPC, having evidence of locally advanced disease as per the TNM (tumor-node-metastasis) staging framework of the American Joint Cancer Committee (AJCC) 8^th^ edition (T1-2N1-3M0 or T3-4aN0-3M0) [14], having no prior history of cancer, not having received systemic chemotherapy or radiotherapy (RT) to the head and neck, receiving definitive (CCRT) with at least one course of concurrent chemotherapy, and having available electronic records of RT dosimetry, oral examination, and complete blood counts before the commencement of CCRT.

Patients with a baseline maximum mouth opening (MMO) of ≤35 mm were considered ineligible, as this value indicates trismus per Dijkstra's widely accepted definition for cancer patients. [15] Individuals presenting with missing maxillary and/or mandibular central incisors, prior TMJ surgery, TMJ ankylosis, head and neck trauma, muscle-related pain or myofascial pain syndrome, or primary tumor or lymph node invasion of the masticatory muscles were also excluded from the research. To minimize the bias introduced by these confounding variables, patients who had used anti-inflammatory drugs and steroids within 30 days before beginning CCRT and those with systemic inflammatory conditions, collagen vascular diseases, blood transfusions, and hemoglobinopathies were also excluded.

### 2.3. MMO Measurements and Determination of PIV, Hb, and PIV/Hb Values

All assessments before CCRT were conducted within two weeks of the scheduled therapy start date. To determine the trismus status, the Therabite® motion scale (Atos Medical AB, Hörby, Sweden) was used to measure the distance between the upper and lower central incisors on the same side, with the mouth voluntarily being wide open [16]. Each patient's MMO measurements were recorded before CCRT and at various intervals post-CCRT (1, 3, 6, 9, 12, 18, and 24 months), as well as during scheduled visits or as necessary. Trismus was diagnosed according to the criteria set forth by Dijkstra et al., [15] with a cutoff of ≤35 mm, based on MMO measurements taken before and after CCRT, with each patient receiving three consecutive measurements and the mean of these readings being reported.

We obtained the pre-CCRT Hb (g/dL) readings from the complete blood count test results acquired on the first day of the CCRT start. Using the original formula, the pretreatment PIV was calculated using the following formula: PIV = [P × M × N] ÷ L, where P, M, N, and L are the pretreatment platelet, monocyte, neutrophil, and lymphocyte counts, respectively, obtained from the same test utilized for Hb readings. [9] Using the pre-CCRT PIV and Hb measurements, the novel PIV/Hb index was calculated using the following formula: PIV/Hb = PIV ÷ Hb.

### 2.4. Treatment Protocol

Our institution's standard of care for treating LA-NPC is the simultaneous integrated boost intensity-modulated RT (SIB-IMRT). To define the target volumes for RT, we use pretreatment coregistered computed tomography (CT), 18-fluorodeoxyglucose-positron emission tomography (PET)-CT (FDG-PET-CT), and/or magnetic resonance imaging (MRI) scans of the affected primary site and the entire neck. [17] The specific RT doses for each target volume were defined based on previous literature. [17] To provide an overview, the doses administered to the planning target volumes (PTVs) were 70.0 Gy for high-risk PTVs, 59.5 Gy for intermediate-risk PTVs, and 54.0 Gy for low-risk PTVs. The treatment was delivered using single daily fractions throughout 33 days. [17] Depending on patient tolerance to chemotherapy, 1 to 3 cycles of cisplatin and 5-fluorouracil combination were delivered concurrently with RT every 21 days. For all patients, adjuvant treatment was recommended, which included two cycles of the same chemotherapy regimen. The patients in this study received treatment overseen by the same adept radiation oncologist (E.T.). The diagnosis, staging, radiation therapy planning, and treatment administration have remained the same, except for adjustments made in line with updated pertinent guidelines.

### 2.5. Statistical Analysis

The primary objective of this retrospective study was to examine whether there is a meaningful relationship between pre-CCRT PIV/Hb values and RIT rates. The final day of CCRT was the basis for computing the timeframes for RIT diagnosis. Patient data were censored in case of a diagnosis of RIT, loss to follow-up, or death. Categorical data were presented using percentage frequency distributions, while continuous variables were represented using medians, means, and ranges, as required. Using receiver operating characteristic (ROC) curve analysis, we estimated the cutoff(s) for continuous variables that, if present, could split the entire research cohort into two groups with significantly different outcomes, both before and after the CCRT. Logistic regression analysis was utilized to assess the independent significance of each variable in the multivariate analysis. All tests were two-tailed, with a *p* value of <0.05 deemed statistically significant.

## 3. Results

In this research, we retrospectively analyzed the data from 228 individuals diagnosed with LA-NPC. As shown in Table 1, the median age of the patients was 56.5 years, with an age range of 18 to 76 years. Of the total patients, 159 (69.7%) were male. The proportion of patients who had previously used tobacco or alcohol was 62.7% and 32.0%, respectively. Most patients had advanced disease stages of T3-4 (*N* = 166, 72.8%) or N2-3 (*N* = 183, 80.3%). Anemia was found in 48.2% (*N* = 110) of patients, as defined by the World Health Organization's (WHO) criteria of Hb levels <12.0 g/dL in women and <13.0 g/dL in men [18]. Additionally, seniors aged 65 and above comprised approximately 24.6% of all cases, according to the WHO's definition of elderly individuals. [19].

The final assessments conducted after CCRT revealed an absolute 3.1 mm (7.5%) decrease in MMO measures from a pre-CCRT median of 41.4 mm (range: 37.8–46.8 mm) to a post-CCRT median of 38.3 mm (range: 25–44 mm) (Tables 1 and 2). Among the 228 patients who underwent CCRT, 46 patients (20.2%) were diagnosed with RIT, as defined by MMO ≤35 mm by Dijkstra and colleagues for cancer patients [15]. The median time between the CCRT and RIT diagnosis was 10 months (range: 6–18 months).

**Figure 1:**
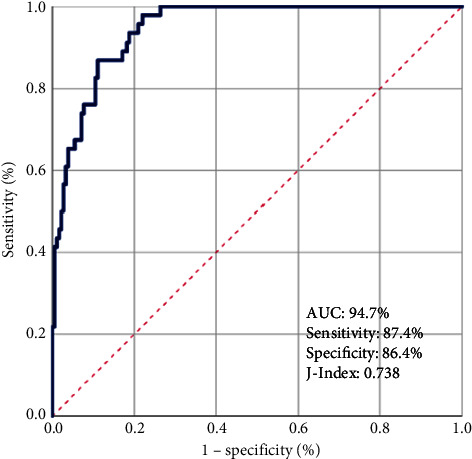
The outcomes of a receiver operating characteristic (ROC) curve analysis examining the correlation between the combined pretreatment pan-immune-inflammatory index and hemoglobin levels (PIV/Hb) and post-CCRT radiation-induced trismus rates following concurrent chemoradiotherapy [PIV/Hb cutoff: 68.4 g/dL; area under the curve (AUC): 94.7%; sensitivity = 87.4%; specificity = 86.4%; J-index = 0.738].

The median Hb level before CCRT for the entire group was 12.65 g/dL (range: 8.96–17.30 g/dL). We conducted an ROC curve analysis to investigate potential correlations between RIT incidence rates and baseline Hb levels. The optimal cutoff was found to be 11.9 g/dL, indicating a meaningful relationship [area under the curve (AUC): 82.7%; sensitivity: 72.9%; specificity: 71.7%; J-index: 0.556] (Figure 1). As a result, we divided the study population into two groups: Group 1 (Hb ≥ 12 g/dL; *N* = 141) and Group 2 (Hb < 12.0 g/dL; *N* = 87). Cross-tabulation with the Chi-square test showed that the incidence of RIT was significantly higher in the Hb < 12 g/dL group compared to the Hb ≥ 12 g/dL group [34.5% vs. 7.1%; hazard ratio (HR): 4.86; *P*  <  0.001] (Table 3).

Similarly, ROC curve analysis was utilized to identify probable significant associations between pre-CCRT PIV levels and RIT rates, yielding 813 as the optimal cutoff value (AUC: 92.7%; sensitivity: 84.8%; and specificity: 83.7%; J-index: 0.685). Consequently, this cutoff separated the whole research population into two groups: Group 1: PIV ≤813 (*N* = 160) and Group 2: PIV >813 (*N* = 68). Despite having comparable distributions of all variables, patients in Group 2 had a significantly higher incidence of RIT than those in Group 1 (57.2% vs. 4.2%; HR: 13.6; *P*  <  0.001), as shown in Table 3.

Based on our findings, which showed that pre-CCRT PIV and Hb levels may effectively predict the risk of RIT, we aimed to create a new composite index by combining PIV values with Hb values. We intended to investigate the potential of developing a more precise composite biomarker than each component on its own (PIV/Hb = PIV ÷ Hb). Thus, we used ROC curve analysis to identify a pre-CCRT PIV/Hb level cutoff point that could divide patients into two distinct RIT risk groups. Our analysis concluded that the optimal cutoff point was 68.4 (AUC = 94.7%; sensitivity = 87.4%; specificity = 86.4%; J-index = 0.738), rounded to 68 for ease of use in subsequent analysis (Figure 1). Consequently, we categorized all patients into two final groups using this cutoff value: Group 1: PIV/Hb ≤ 68 (*N* = 160) and Group 2: PIV/Hb > 68 (*N* = 68). Comparisons between the two groups demonstrated that the RIT incidence was higher in the PIV/Hb > 68 group than its PIV/Hb ≤ 68 counterparts (58.8% vs. 3.8%; HR: 17.4; *P*  <  0.001) (Table 3).

According to the univariate analyses (Table 3), there were significant associations between RIT incidences and various pre-CCRT groups. Namely, MMO (31.3% vs. 9.5% for ≥41.4 mm; *P*  <  0.001), masticatory apparatus dose (MAD) V58 (44.3% vs. 5% for V58 > 32%; *P*  <  0.001), anemia status (37.3% vs. 4.2% for absence; *P*  <  0.001), pre-CCRT Hb (34.5% vs. 7.1 for >12 g/dL, *P*  <  0.001), pre-CCRT PIV (57.4% vs. 4.2% for PIV ≤ 813, *P*  <  0.001), and pre-CCRT PIV/Hb (58.8% vs. 3.8% for PIV/Hb ≤ 68, *P*  <  0.001), with former groups exhibiting higher RIT rates compared to their respective comparator groups. Multivariate logistic regression analyses confirmed that each of the six characteristics was an independent and significant predictor of RIT in patients with LA-NPC who underwent definitive CCRT (*P*  <  0.05 for each), as shown in Table 3. Finally, we analyzed the relative predictive powers of Hb, PIV, and PIV/Hb parameters for RIT through Spearman correlation analysis. Our analysis showed that PIV/Hb had the highest correlation (rs = 0.82) with rates, compared to PIV (rs = 0.74) and Hb (rs = 0.51) parameters.

## 4. Discussion

The primary objective of this retrospective study was to assess the viability of using pre-CCRT PIV/HB levels as a biomarker for predicting the occurrence of RIT in patients with LA-NPC. The most striking result of the current retrospective study was that patients with PIV/Hb > 68 before CCRT had a significantly higher incidence of RIT than those with PIV/Hb ≤ 68 (58.8% vs. 3.8%; HR: 17.4; *p* <  0.001). The Spearman correlation analysis revealed that the PIV/Hb parameter had the strongest correlation (*r*_*s*_ = 0.82) with RIT rates, compared to PIV (*r*_*s*_ = 0.74) and Hb (*r*_*s*_ = 0.51). Other remarkable findings were pre-CCRT MMO (31.3% vs. 9.5% for ≥41.4 mm group; *p* < 0.001), MAD V58 (44.3% vs. 5% for V58 ≤ 32% group; *p* <  0.001), anemia status (37.3% vs. 4.2% for the nonanemic group; *p* <  0.001), pre-CCRT Hb (34.5% vs. 7.1 for Hb > 12 g/dL group, *p* <  0.001), and pre-CCRT PIV (57.4% vs. 4.2% for PIV ≤ 813 groups; *p* <  0.001), with former groups exhibiting higher RIT rates than their comparator groups.

We examined various pre-CCRT factors that could be linked to RIT rates before CCRT and found that Hb levels (*p* <  0.001), anemia status (*p* <  0.001), MMO measures (*p* <  0.001), MAD V58 (*p* <  0.001), and PIV (*p* <  0.001) all had significant correlations with RIT rates. Recently, Somay et al. [12] identified an ideal Hb cutoff of 12 g/dL, which helped predict RIT rates in LA-NPC patients with high accuracy (41.9% for Hb ≤ 12 g/dL vs. 7.3% for Hb > 12 g/dL, *p* <  0.001). Similarly, RIT rates were higher in the anemic group than in the nonanemic group as per WHO's anemia definition (37.3% vs. 4.2%; *p* <  0.001). Fewer studies have been dedicated to examining the impact of MMO prior to treatment on the rates of RIT compared to other influencing factors. [20–22] Kraaijenga et al. observed that an MMO measurement of <46 mm before treatment correlated with an elevated risk of RIT. [20] Similarly, Owosho et al. noted a significantly higher incidence of RIT among individuals with an MMO measurement of <40 mm before RT compared to those with an MMO measurement >40 mm. [21] Supporting these findings, Somay et al. identified that among 198 LA-NPC patients undergoing CCRT, the subgroup with a mean MMO of ≤40.7 mm before CCRT exhibited a higher RIT rate compared to the subgroup with a mean MMO >40.7 mm (42.2% vs. 11.9% for the >40.7 mm subgroup; HR: 3.47; *p* <  0.001). [8] Previous research found that the mean and maximum dosages of MAD and TMJ are dosimetric parameters that affect RIT rates. [23, 24] Higher V50 values were associated with significantly increased RIT rates in a study by Kraaijenga and colleagues. [20] Similarly, our previous research showed that patients with MAD V58 Gy ≥ 32% had a much higher incidence of RIT compared to those with a lower dose (44.3% vs. 5% for V58 Gy < 32%; *p* <  0.001). [12] Therefore, our findings, along with those of other researchers, suggest that all four of these parameters significantly affect RIT rates, emphasizing the importance of these factors in the development of RIT.

The most notable discovery in our present research was that patients with PIV/Hb > 68 had a considerably higher occurrence of RIT than their PIV/Hb ≤ 68 counterparts (58.8% vs. 3.8%; HR: 17.4; *p* <  0.001). Based on the robust and separate connections found in our previous research between RIT rates and pre-CCRT Hb and PIV values (*p* <  0.001 for each), we were inspired to examine the impact of the PIV/Hb ratio as a new and potentially more effective biomarker in this group of patients. [12, 25] In previous studies, radiation-induced fibrosis, the basis of RIT, has been shown to develop as a late complication of RT and was activated by hypoxia and its key mediators such as hypoxia-inducible factors and other inflammatory mediators (transforming growth factor-beta1, tumour necrosis factor *α*, etc.) [25]. The comprehensive nature of PIV as a systemic immune and inflammation marker, which incorporates almost all cells functioning in these processes in its unique formula, enhances its effectiveness as a biomarker in predicting the development of fibrosis and related RIT after RT or CCRT [9]. Therefore, it was reasonable to assume that the RIT rate could be estimated more accurately using a combination of PIV/Hb than by using Hb and PIV separately. The Spearman correlation analysis confirmed this assumption, indicating that the PIV/Hb parameter exhibited the strongest correlation (*r*_*s*_ = 0.82) with RIT rates, surpassing the correlations of PIV (*r*_*s*_ = 0.74) and Hb (*r*_*s*_ = 0.51). This finding aligns with the earlier research conducted by Somay et al. on 223 LA-NPC patients. [12] In their study, the authors established a strong and separate correlation between a higher occurrence of RIT during CCRT and the existence of Hb levels <12.0 g/dL (41.9%) or anemia (37.6%). Our research findings are also corroborated by a recent study, which revealed that a pre-CCRT PIV >830 was strongly correlated with a higher prevalence of RIT compared to its PIV ≤830 counterpart (60.3% vs. 5%; HR 5.79; *p* <  0.001). 26 Therefore, our results and backing prior study findings suggest that the new PIV/Hb index is a more comprehensive and robust predictor of post-CCRT RIT incidence rates in LA-NPC and, presumably, other head and neck malignancies. [7, 8, 12] However, further research is desperately needed to corroborate these results and ascertain the applicability of the novel PIV/Hb index in predicting RIT rates in other head and neck cancers subjected to RT or CCRT.

The precise interaction and pathophysiological mechanisms linking high PIV values to increased rates of RIT have yet to be fully elucidated. However, PIV is an index that encompasses blood-borne neutrophils, platelets, monocytes, and lymphocytes. Therefore, an elevation in PIV is thought to signify heightened systemic inflammation and compromised immunity. [9] Neutrophils and platelets can activate fibrosis by releasing inflammatory mediators such as the vascular endothelial growth factor, tumor necrosis factor-*α*, and interleukin-10 in response to systemic inflammation. [23, 26] Recent research has confirmed the significance of composite biomarkers, such as NLR and HPR, in predicting the prevalence of RIT. [7, 8] Moreover, these studies have provided evidence in support of the hypothesis that RT stimulates the production and release of proinflammatory cytokines. It is pertinent to mention that the PIV/Hb index analyzed in this current research comprises both NLR and HPR indexes. The activation of inflammatory mediators and induction of fibrosis by monocytes are known factors contributing to progressive muscular dystrophy, including the masticatory muscles. [27] As such, it is plausible to consider the potential crucial involvement of monocytes in the pathogenesis and progression of RIT. Furthermore, Sharma et al. [28] have documented that diminished hemoglobin levels initiate a self-perpetuating cycle of local hypoxia, ischemia, and fibrosis, thereby amplifying harmful inflammation in the masticatory structures and facilitating the development of RIT. This finding aligns with the proposition by Lo et al., suggesting that reduced hemoglobin levels may impede the proper tissue repair process in the affected masticatory apparatus. [29] This delay can result in a persistent hypoxic state, which in turn can stimulate inflammatory and fibrosis cascades. While the exact mechanisms involved may be more complex, a high PIV/Hb index indicates an exaggerated systemic inflammatory response, compromised immunity, and/or tissue hypoxia, all of which play crucial roles in all phases of RIT pathogenesis (Table 4).

The findings of this study should be interpreted with caution due to certain limitations. Firstly, the results are based on a retrospective cohort study from a single institution, which may introduce unanticipated biases. Therefore, further follow-up studies are required to validate the presented results. Secondly, the fluctuating nature of Hb levels during and after treatment means that using the WHO's anemia criteria and a single pre-CCRT hemoglobin cutoff may not accurately reflect the incidence difference between RIT and the rates of this complication. The same applies to the PIV measures used in this study. Third, although Epstein-Barr virus (EBV) infection may cause hematological abnormalities, mainly atypical lymphocytosis, and mild decreases in platelet counts, the EBV status was not correlated with the RIT outcomes in our study. [34] And fourth, it is imperative to acknowledge that this study's utilization of peripheral blood Hb measures is an indirect approach to assessing tissue hypoxia as we did not conduct any direct evaluations of vascular abnormalities, in vivo oxygen measurements, or blood measures of the vascular endothelial growth factor or hypoxia-inducible factor 1-alpha. Additionally, we did not correlate our results with immune-inflammatory and fibrosis indicators, such as tumor necrosis factor-alpha, interleukin-1, interleukin-6, interleukin-8, basic fibroblast growth factor, or transforming growth factor-beta. Therefore, the results presented in this study should be considered hypothetical rather than conclusive remarks, requiring further confirmatory research before its applicability to routine oncology clinics.

## 5. Conclusion

The current research findings suggest that the novel PIV/Hb index before CCRT for LA-NPC is a potent and independent factor for stratifying these patients into subgroups with significantly different RIT rates. If the cost-effective and simple-to-measure PIV/Hb index proves to be a reliable indicator of RIT rates in future large-scale studies, it could assist in identifying high-risk patients before treatment and promptly implementing preventive measures.

## Figures and Tables

**Table 1:** Baseline and treatment characteristics of the whole study cohort per hemoglobin, pan-immune-inflammation value, and combining pan-immune-inflammation value and hemoglobin group.

Characteristics	All patients (*N* = 228)	Hb ≤ 12 (*N* = 87)	Hb > 12 (*N* = 141)	*p*	PIV>813 (*N* = 68)	PIV ≤813 (*N* = 160)	*p*	PIV/Hb > 68 (*N* = 68)	PIV/Hb ≤ 68 (*N* = 160)	*p*
Median age, years (range)	56.5 (18–76)	57 (18–76)	56 (18–76)	0.43	58.5 (18–76)	56 (18–76)	0.20	59 (18–76)	56 (18–76)	0.62

*Age group, years*										
≤56.5	114 (50)	43 (49.4)	71 (50.3)	1.00	29 (42.6)	85 (53.1)	0.19	30 (44.1)	84 (52.5)	0.24
>56.5	114 (50)	44 (50.6)	70 (49.7)		39 (57.4)	75 (46.9)		38 (55.8)	76 (47.5)	

*Gender, N (%)*										
Female	69 (30.3)	30 (34.5)	39 (27.6)	0.30	25 (36.7)	44 (27.5)	0.21	25 (36.7)	44 (27.5)	0.16
Male	159 (69.7)	57 (65.5)	102 (72.4)		43 (56.3)	116 (72.5)		43 (63.3)	116 (72.5)	

*Smoking status, N (%)*										
Yes	143 (62.7)	32 (36.8)	53 (37.5)	1.00	41 (60.3)	102 (63.7)	0.66	39 (57.4)	104 (65.0)	0.27
No	85 (37.3)	55 (63.2)	88 (62.5)		27 (39.7)	58 (36.3)		29 (42.6)	56 (35.0)	

*Alcohol consumption, N (%)*										
Yes	73 (32)	59 (67.8)	96 (68.0)	1.00	15 (22.1)	58 (36.3)	0.04	16 (23.5)	57 (35.6)	0.07
No	155 (68)	28 (32.2)	45 (32.0)		53 (77.9)	102 (63.7)		52 (76.5)	103 (64.4)	

*Median pre-CCRT MMO, N (range-mm)*										
	41.4 (37.8–46.8)	41 (37.8–46.0)	41.7 (38.0–46.8)	0.32	40.5 (38–45)	41.8 (37.8–46.8)	0.12	40.5 (38–45)	41.8 (37.8–46.8)	0.069

*Pre-CCRT MMO group, N (%) (mm)*										
<41.4	112 (49.1)	51 (58.6)	61 (43.2)	0.029	46 (67.6)	66 (41.3)	<0.001	44 (64.7)	68 (42.5)	0.002
≥41.4	116 (50.9)	36 (41.4)	80 (56.8)		22 (32.4)	94 (58.7)		24 (35.3)	92 (57.5)	

*T-stage, N (%)*										
1–2	62 (27.2)	25 (28.7)	37 (26.2)	0.83	17 (25.0)	45 (28.1)	0.59	19 (27.9)	43 (26.9)	0.74
3–4	166 (72.8)	62 (71.3)	104 (73.8)		51 (75.0)	115 (71.9)		49 (72.1)	107 (73.1)	

*N-stage, N (%)*										
0–1	45 (19.7)	15 (17.2)	30 (21.2)	0.26	18 (26.5)	27 (16.9)	0.31	16 (23.5)	29 (18.1)	0.21
2–3	183 (80.3)	72 (82.8)	111 (78.8)		50 (73.5)	133 (83.1)		52 (76.5)	131 (81.9)	

*WHO histology, N (%)*										
1	0 (0.0)	0 (0.0)	0 (0.0)	0.88	0 (0.0)	0 (0.0)	0.91	0 (0.0)	0 (0.0)	0.82
2	25 (10.9)	9 (10.3)	16 (11.3)		8 (11.8)	17 (10.6)		7 (10.3)	18 (11.3)	
3	203 (89.1)	78 (89.7)	125 (88.7)		60 (88.2)	143 (89.4)		61 (89.7)	124 (88.7)	

HB: hemoglobin; PIV: pan-immune-inflammation value; CCRT: concurrent chemoradiotherapy; MMO: maximum mouth opening; T: tumor; N: node; WHO: World Health Organization.

**Table 2:** Treatment characteristics of the entire study cohort per hemoglobin, pan-immune-inflammation value, and combining pan-immune-inflammation value and hemoglobin group.

Characteristics	All patients (*N* = 228)	Hb ≤ 12 (*N* = 87)	Hb > 12 (*N* = 141)	*p*	PIV > 813 (*N* = 68)	PIV ≤ 813 (*N* = 160)	*p*	PIV/Hb > 698 (*N* = 68)	PIV/Hb ≤ 698 (*N* = 160)	*p*
Mean MAD; gy (range)	36.4 (12.3–67.5)	35.7 (14.3–67.2)	37.3 (12.3–66.9)	0.65	37.3 (13.8–67.6)	35.4 (12.3–65.9)	0.63	35.9 (13.7–66.4)	37.1 (12.3–67.5)	0.78
Mean MMAD; gy (range)	46.3 (28.3–79.0)	45.6 (28.1–78.7)	48.2 (28.2–78.9)	0.31	45.4 (28.6–79.0)	46.9 (28.3–77.4)	0.49	45.8 (28.9–79.0)	47.6 (28.3–77.7)	0.62

*MAD V58 Gy group, N (%)*										
<32%	140 (61.4)	53 (61.0)	87 (61.7)	0.74	38 (55.9)	102 (63.8)	0.67	41 (60.3)	99 (61.9)	0.84
≥32%	88 (38.6)	34 (39.0)	54 (38.3)		30 (44.1)	58 (36.2)		27 (39.7)	61 (38.1)	

*Median time from RIT to CCRT, month (range)*										
	10 (6–18)	10 (6–15)	12 (8–16)	0.28	10 (6–12)	10 (7–18)	0.14	10 (6–16)	10 (7–14)	0.24

*Median post-CCRT MMO, mm (range)*										
	38.3 (25.9–44.0)	37 (35.9–44)	39 (38.0–44.0)	0.028	35 (25.9–42.5)	39 (32.7–44.0)	<0.001	34.6 (25.9–42.5)	39 (32.7–44.0)	<0.001

*Post-CCRT RIT, N (%)*										
Absent	182 (79.8)	51 (58.6)	131 (92.9)	<0.001	29 (42.6)	153 (95.6)	<0.001	28 (41.2)	154 (96.3)	<0.001
Present	46 (20.2)	36 (41.4)	10 (7.1)		39 (57.4)	7 (4.4)		40 (58.8)	6 (3.7)	

*Concurrent chemotherapy cycles, N (%)*										
1										
2–3	45 (19.7)	17 (19.5)	28 (19.8)	0.49	15 (22.1)	30 (18.8)	0.82	16 (23.5)	29 (18.1)	0.39
	183 (80.3)	70 (80.5)	113 (80.2)		53 (77.9)	130 (81.2)		52 (66.5)	131 (81.9)	

*Adjuvant chemotherapy cycles, N (%)*										
0										
1–2	56 (24.6)	24 (27.6)	32 (22.7)	0.37	17 (25.0)	39 (24.4)	0.78	19 (27.9)	37 (23.1)	0.72
	172 (75.4)	63 (72.4)	109 (77.3)		51 (75.0)	121 (65.6)		49 (72.1)	123 (76.9)	

HB: hemoglobin; PIV: pretreatment pan-immune-inflammatory value; MAD: masticatory apparatus dose; Gy: gray; pre: pretreatment; CCRT: concurrent chemoradiotherapy; MMO: maximum mouth opening; mm: millimeter; RIT: radiation-induced trismus.

**Table 3:** Radiation-induced trismus outcomes of the entire study group.

Characteristics	All patients (*N* = 228)	RIT (%) (*N* = 46)	Univariate *p* value	Multivariate *p* value	HR (95% CI)
*Age group, years, N (%)*					
≤57	114 (50)	16.7	0.284	0.33	1.14 (0.87–1.41)
>57	114 (50)	23.1			

*Gender, N (%)*					
Female	69 (30.3)	24.6	0.248	0.31	1.12 (0.91–1.34)
Male	159 (69.7)	18.2			

*Smoking status, N (%)*					
No	143 (62.7)	18.2	0.39	0.45	1.09 (0.79–1.42)
Yes	85 (37.3)	23.5			

*Alcohol consumption, N (%)*					
No	155 (68)	22.5	0.218	0.26	1.16 (0.93–1.48)
Yes	73 (32)	14.7			

*Pre-CCRT MMO group, N (%)*					
<41.4 mm	112 (49.1)	31.3	<0.001	0.002	3.42 (2.17–4.67)
≥41.4 mm	116 (50.9)	9.5			

*T-stage group, N (%)*					
1–2	62 (27.2)	16.1	0.28	0.37	1.08 (0.92–1.24)
3–4	166 (72.8)	21.7			

*N-stage group, N (%)*					
0–1	45 (19.7)	20.0	0.84	0.91	1.02 (0.96–1.07)
2–3	183 (80.3)	20.2			

*Pre-CCRT Hb group, N (%)*					
≤12 g/dL	87 (38.2)	34.5	<0.001	<0.001	4.86 (3.18–6.67)
>12 g/dL	141 (61.8)	7.1			

*Pre-CCRT PIV group, N (%)*					
PIV > 813	68 (29.8)	57.4	<0.001	<0.001	13.6 (8.79–21.34)
PIV ≤ 813	160 (70.2)	4.2			

*PIV/Hb group, N(%)*					
PIV/Hb > 68	68 (29.8)	58.8	<0.001	<0.001	17.4 (12.32–23.81)
PIV/Hb ≤ 68	160 (70.2)	3.8			

*Anemia, N (%)*					
Absent	118 (51.8)	4.2	<0.001	<0.001	8.19 (5.81–10.61)
Present	110 (48.2)	37.3			

*Concurrent chemotherapy cycles, N (%)*					
1					
2–3	45 (19.7)	17.8	0.54	0.63	1.12 (0.69–1.43)
	183 (80.3)	20.7			

*Adjuvant chemotherapy cycles, N (%)*					
0					
1–2	56 (25.6)	23.2	0.67	0.74	1.09 (0.88–1.33)
	172 (74.4)	19.2			

*MAD V58 Gy group, N (%)*					
<32%	140 (61.4)	5.0	<0.001	<0.001	8.74 (5.38–11.12)
≥32%	88 (38.6)	44.3			

HB: hemoglobin; PIV: pretreatment pan-immune-inflammatory value; HR: hazard ratio; pre: pretreatment; CCRT: concurrent chemoradiotherapy; Gy: gray; V: volume; MAD: masticatory apparatus dose; MMO: maximum mouth opening; mm: millimeter; T: tumor; N: node; RIT: radiation-induced trismus.

**Table 4:** The studies about biomarkers related to radiation-induced rates.

Author	Year	Study design	Biomarker	RIT rates (%)	*p* value	Conclusion
Somay et al. [8]	2022	Retrospective	Hemoglobin-to-platelet ratio (HPR)	HPR ≤ 0.54 : 34.1% HPR > 0.54 : 12.8%	<0.001	HPR > 0.54 is a robust risk factor for elevated RIT rates

Somay et al. [7]	2022	Retrospective	Neutrophil-to-lymphocyte ratio (NLR)	NLR > 2.7 : 35.2% LR ≤ 2.7 : 5.8%	<0.001	NLR > 2.7 is a strong predictor of increased RIT incidence

Topkan et al. [30]	2023	Retrospective	Host index [H-index: (*N* × M) ÷ (Albumin × L × Hb) × 100]	H-index > 5.5 : 31.8% H-index ≤ 5.5 : 5.9%	<0.001	Pre-C-CRT H-index > 5.5 is associated with significantly increased RIT rates
Somay et al. [31]	2023	Retrospective	Systemic inflammation score [SIS: albumin and lymphocyte-to-monocyte ratio (LMR)]	SIS-0: 5.4% SIS-1: 11.7% SIS-2: 45.0%	<0.001	SIS is a dependable biomarker-based system that can accurately predict the rates of RIT
Somay et al. [32]	2023	Retrospective	Combined hemoglobin-to-platelet ratio and maximum mouth opening index (HPR-MMO index)	Low-risk: 10.2% Intermediate-risk: 19.2% High-risk: 59.4%	<0.001	The combined HPR-MMO scoring system efficiently stratifies patients into three RIT risk groups
Somay et al. [25]	2024	Retrospective	Pan-immune-inflammation value [PIV = (platelets × monocytes × neutrophils) ÷ lymphocytes)]	PIV > 830 : 60.3 PIV ≤ 830 : 5.0%	<0.001	RIT was significantly more prevalent in the PIV > 830 cohort
Somay et al. [12]	2024	Retrospective	Hemoglobin	Hb ≤ 12 g/dL: 41.9 Hb > 12 g/dL: 7.3%	<0.001	RIT was substantially more frequent in the hb ≤ 12 g/dL group
Somay et al. [33]	2024	Retrospective	Global immune-nutrition-inflammation index [GINI = (CRP × M × P × N) ÷ (Albumin × L)]	GINI < 1424 : 43.3% GINI < 1424 : 9.3%	<0.001	A pre-CCRT GINI ≥ 1424 is connected to a significantly higher RIT incidence

RIT: radiation-induced trismus; N: neutrophil; *M*: monocyte; P: platelet; CCRT: concurrent chemoradiotherapy; L: lymphocytes; CRP: C- reactive protein; pre: pretreatment.

## Data Availability

For researchers who satisfy the criteria for access to sensitive data, the datasets utilized and/or analyzed during the current study are accessible from the Baskent University Department of Radiation Oncology Institutional Data Access.

## Abbreviations

PIV/Hb: The pan-immune-inflammatory index and hemoglobin levels
RIT: Radiation-induced trismus
LA-NPC: Locally advanced nasopharyngeal cancer
CCRT: Concurrent chemoradiotherapy
MMO: Maximum mouth openings
ROC: Receiver operating characteristic
HPR: Hemoglobin-to-platelet ratio
NLR: Neutrophil-to-platelet ratio
PIV: Pan-immune-inflammation value
TMJ: Temporomandibular joint
TMD: Temporomandibular disorder
Hb: Hemoglobin
MRI: Magnetic resonance imaging
ECOG: Eastern Cooperative Oncology Group
PTVs: Planning target volumes
AUC: Area under the curve
MAD: Masticatory apparatus dose
HIFs: Hypoxia-inducible factors
VGEF: Vascular endothelial growth factor
TNF-*α*: Tumor necrosis factor-*α*
IL10: Interleukin-10.

## Additional Points

*Key points*. (1) Radiation-induced trismus (RIT) affects up to 42% of head and neck cancer patients. (2) Radiation-induced fibrosis, the triggering factor of RIT, manifests as a delayed consequence of radiotherapy and is thought to be initiated by hypoxia and its principal mediators. (3) To evaluate the utility of a new index on the prevalence of RIT in patients receiving concurrent chemoradiotherapy for locally advanced nasopharyngeal cancer, we integrated the pan-immune-inflammatory index and hemoglobin levels (PIV/Hb). (4) 228 LA-NPC patients were divided into two groups according to the cutoff value of 68.4 g/dL determined in the receiver operating characteristic curve analysis. (5) The higher RIT rates in this patient group were correlated with elevated pretreatment levels of the novel PIV/Hb index.

## References

[1] Blanchard P., Lee A., Marguet S. (2015). Chemotherapy and radiotherapy in nasopharyngeal carcinoma: an update of the MAC-NPC meta-analysis. *The Lancet Oncology*.

[2] Rocha P. H. P., Reali R. M., Decnop M. (2022). Adverse radiation therapy effects in the treatment of head and neck tumors. *Radio Graphics*.

[3] Brook I. (2020). Late side effects of radiation treatment for head and neck cancer. *Radiation Oncology Journal*.

[4] Strojan P., Hutcheson K. A., Eisbruch A. (2017). Treatment of late sequelae after radiotherapy for head and neck cancer. *Cancer Treatment Reviews*.

[5] Chang D. S., Lasley F. D., Das I. J., Mendonca M. S., Dynlacht J. R. (2021). Normal tissue radiation response. *Basic Radiotherapy Physics and Biology*.

[6] Pattani N., Sanghera J., Langridge B. J., Frommer M. L., Abu-Hanna J., Butler P. (2024). Exploring the mechanisms behind autologous lipotransfer for radiation-induced fibrosis: a systematic review. *PLoS One*.

[7] Somay E., Yilmaz B., Topkan E., Kucuk A., Pehlivan B., Selek U. (2023). Initial neutrophil-to-lymphocyte ratio predicts radiation-induced trismus in parotid gland cancer. *Oral Diseases*.

[8] Somay E., Yilmaz B., Topkan E. (2023). Hemoglobin-to-platelet ratio in predicting the incidence of trismus after concurrent chemoradiotherapy. *Oral Diseases*.

[9] Fucà G., Guarini V., Antoniotti C. (2020). The Pan-Immune-Inflammation Value is a new prognostic biomarker in metastatic colorectal cancer: results from a pooled-analysis of the Valentino and TRIBE first-line trials. *British Journal of Cancer*.

[10] Guo H., Huang J., Liang Y., Wang D., Zhang H. (2022). Focusing on the hypoxia-inducible factor pathway: role, regulation, and therapy for osteoarthritis. *European Journal of Medical Research*.

[11] Karthik H., Nair P., Gharote H. P., Agarwal K., Ramamurthy Bhat G., Kalyanpur Rajaram D. (2012). Role of hemoglobin and serum iron in oral submucous fibrosis: a clinical study. *The Scientific World Journal*.

[12] Somay E., Yilmaz B., Topkan E., Pehlivan B., Selek U. (2023). Low hemoglobin levels predict increased radiation-induced trismus rates in nasopharyngeal cancer. *Oral Diseases*.

[13] Miranda S. B., Possebon A. P. D. R., Schuster A. J., Marcello-Machado R. M., de Rezende Pinto L., Faot F. (2019). Relationship between masticatory function impairment and oral health-related quality of life of edentulous patients: an interventional study. *Journal of Prosthodontics*.

[14] Hirshoren N., Weinberger J. M., Eliashar R. (2020). Head and neck malignancies classification, the 8th edition of the American Joint Committee on Cancer-what is new?. *Harefuah*.

[15] Dijkstra P. U., Huisman P. M., Roodenburg J. L. (2006). Criteria for trismus in head and neck oncology. *International Journal of Oral and Maxillofacial Surgery*.

[16] Saund D. S., Pearson D., Dietrich T. (2012). Reliability and validity of self-assessment of mouth opening: a validation study. *Bio Medicine Central Oral Health*.

[17] Yilmaz B., Somay E., Selek U., Topkan E. (2021). Pretreatment systemic immune-inflammation index predict needs for teeth extractions for locally advanced head and neck cancer patients undergoing concurrent chemoradiotherapy. *Therapeutics and Clinical Risk Management*.

[18] Cappellini M. D., Motta I. (2015). Anemia in clinical practice-definition and classification: does hemoglobin change with aging?. *Seminars in Hematology*.

[19] World Health Organisation (2012). 10 facts on ageing and the life course. http://www.who.int/features/factfiles/ageing/ageing_facts/en/index.html.

[20] Kraaijenga S. A., Hamming-Vrieze O., Verheijen S. (2019). Radiation dose to the masseter and medial pterygoid muscle in relation to trismus after chemoradiotherapy for advanced head and neck cancer. *Head and Neck*.

[21] Owosho A. A., Pedreira Ramalho L. M., Rosenberg H. I. (2016). Objective assessment of trismus in oral and oropharyngeal cancer patients treated with intensity-modulated radiation therapy (IMRT). *Journal of Cranio-Maxillofacial Surgery*.

[22] Rajalalitha P., Vali S. (2005). Molecular pathogenesis of oral submucous fibrosis-a collagen metabolic disorder. *Journal of Oral Pathology and Medicine*.

[23] Hague C., Beasley W., Garcez K. (2018). Prospective evaluation of relationships between radiotherapy dose to masticatory apparatus and trismus. *Acta Oncologica*.

[24] Morimoto M., Bijl H. P., Van Der Schaaf A. (2019). Development of normal tissue complication probability model for trismus in head and neck cancer patients treated with radiotherapy: the role of dosimetric and clinical factors. *Anticancer Research*.

[25] Somay E., Yilmaz B., Topkan E. (2024). Worth of pan-immune-inflammation value in trismus prediction after concurrent chemoradiotherapy for nasopharyngeal carcinomas. *The International Journal of Biological Markers*.

[26] Triantafyllou E. A., Mylonis I., Simos G., Paraskeva E. (2019). Hypoxia induces pro-fibrotic and fibrosis marker genes in hepatocellular carcinoma cells independently of inflammatory stimulation and the NF-*κ*Β pathway. *Hypoxia*.

[27] Mojumdar K., Liang F., Giordano C. (2014). Inflammatory monocytes promote progression of Duchenne muscular dystrophy and can be therapeutically targeted via CCR2. *EMBO Molecular Medicine*.

[28] Sharma M., Radhakrishnan R. (2017). Limited mouth opening in oral submucous fibrosis: reasons, ramifications, and remedies. *Journal of Oral Pathology and Medicine*.

[29] Lo L. J., Lin C. L., Chen Y. R. (2008). A device for temporomandibular joint exercise and trismus correction: design and clinical application. *Journal of Plastic, Reconstructive and Aesthetic Surgery*.

[30] Topkan E., Somay E., Yilmaz B., Pehlivan B., Selek U. (2023). Valero’s host index is useful in predicting radiation-induced trismus and osteoradionecrosis of the jaw risks in locally advanced nasopharyngeal carcinoma patients. *Bio medicine Cancer*.

[31] Somay E., Sezen D., Selek U., Besen A. A., Mertsoylu H., Topkan E. (2023). Systemic inflammation score for predicting radiation-induced trismus and osteoradionecrosis of the jaw rates in locally advanced nasopharyngeal carcinoma patients. *Int Journal of Hem Oncology*.

[32] Somay E., Yilmaz B., Topkan E., Kucuk A., Pehlivan B., Selek U. (2023). The predictive usefulness of the novel combined hemoglobin-to-platelet ratio and maximum mouth opening index on prevalence of radiation-induced trismus in patients with nasopharyngeal cancer. *Head and Neck*.

[33] Somay E., Topkan E., Bascil S. (2024). Global Immune-Nutrition-Inflammation Index as a novel comprehensive biomarker in predicting the radiation-induced trismus rates in locally advanced nasopharyngeal carcinoma patients. *Bio molecule Biomedicine*.

[34] Gonzalez Quintela A., Paez-Guillan E., Campos-Franco J., Alende R. (2023). Hematological abnormalities beyond lymphocytosis during infectious mononucleosis: epstein-barr virus-induced thrombocytopenia. *Mediterranean Journal of Hematology and Infectious Diseases*.

